# Serial echocardiography in preterm infants with bronchopulmonary dysplasia: diagnosing and managing recurrent pulmonary vein stenosis

**DOI:** 10.1515/crpm-2024-0038

**Published:** 2025-03-24

**Authors:** Oishi Sikdar, Mahesh Nanjundappa, Aaron Bell, Matthew Jones, Anne Greenough

**Affiliations:** Department of Neonatology, King’s College London, London, UK; Department of Paediatric Cardiology, Evelina Children’s Hospital, London, UK; Department of Women’s and Children’s Health, Faculty of Life Sciences and Medicine, King’s College London, London, UK

**Keywords:** pulmonary vein stenosis, echocardiography, bronchopulmonary dysplasia

## Abstract

**Objectives:**

To highlight the importance of serial echocardiography in preterm infants with bronchopulmonary dysplasia (BPD) to diagnose recurrent pulmonary vein stenosis (PVS) and understand its contribution to respiratory deteriorations.

**Case presentation:**

A preterm female infant born at 23+5 weeks gestation had numerous complications related to extreme prematurity, including BPD. She was diagnosed with PVS on echocardiogram after experiencing recurrent respiratory deteriorations and pulmonary hypertensive crises. Initial management involved transcutaneous balloon dilatation. A serial echocardiographic programme was implemented, with weekly monitoring of PVS. She suffered multiple respiratory deteriorations secondary to recurrence of PVS, necessitating repeat cardiac catheterisations and transcatheter stenting. Systemic macrolide therapy with sirolimus was used as adjunctive therapy.

**Conclusions:**

Extremely prematurely born infants who develop BPD are at higher risk of recurrent PVS. We demonstrate that serial echocardiographic monitoring facilitates early diagnosis and prompt intervention of PVS. Any respiratory deterioration in such infants should be assessed by an echocardiogram.

## Introduction

Pulmonary vein stenosis (PVS) is characterised by the narrowing of veins carrying oxygenated blood from the lungs to the heart. PVS results in obstructed blood flow to the left atrium, leading to elevated pulmonary venous pressure and subsequent pulmonary venous congestion which in turn leads to right heart dysfunction. Clinically, this manifests as persistent and frequent desaturations, tachypnoea, increased work of breathing and often an unexplained increase in ventilatory and oxygen support.

Prematurely born infants represent up to 40 % of infants with PVS [1]. In this population, primary PVS (defined as no previous congenital heart disease or cardiac intervention) is often associated with bronchopulmonary dysplasia (BPD). Over 30 % of BPD patients have concurrent PVS. Initially it was thought that PVS was a congenital anomaly, but emerging evidence suggests a gradual postnatal evolution influenced by both fetal programming and postnatal insults. Premature infants with PVS may have pulmonary venous congestion, interstitial oedema and cardiac dysfunction mediated, in part by vascular endothelial growth factor (VEGF) and inflammation [2].

There is an increased mortality in infants with severe BPD and PVS [2]. The unpredictable natural history of progressive PVS and its diagnostic challenges, however, underscores the need for further understanding of the neonatal consequences. This case report seeks to highlight the relationship between BPD and PVS and the role that serial echocardiography can play in facilitating management.

## Case presentation

A preterm female infant with a birthweight of 500 g was born at 23+5 weeks of gestation by spontaneous vaginal delivery in the footling breech position. The antenatal course had been uneventful until the mother went into premature labour; she received one dose of antenatal corticosteroids and magnesium sulphate.

The infant was born in poor condition, intubated at 8 min of age and surfactant administered. She was transferred to a Level 3 NICU within 24 h.

The infant received conventional mechanical ventilation (CMV), high frequency oscillation ventilation (HFOV) and volume targeted assist control ventilation (ACV) with an oxygen requirement of between 40 and 50 %. She had a large pulmonary haemorrhage on day three. Inotropic support with dobutamine and noradrenaline was required until day seven. The first echocardiogram on day seven showed a large patent ductus arteriosus (PDA) measuring 2 mm, but a structurally normal heart. A five-day course of paracetamol was given, and a subsequent echocardiogram demonstrated restriction of the duct.

On day 17, the patient was transferred to a surgical unit because of suspicion of necrotising enterocolitis (NEC) and bowel perforation. A second echocardiogram on day 23 undertaken because of a respiratory deterioration demonstrated an unrestricted PDA with a foramen ovale. It was noted that four pulmonary veins connected to the left atrium. She received a further two courses of paracetamol and was started on diuretics. An echocardiogram three weeks later demonstrated a persistent PDA with left to right shunting and, as there was inability to extubate, the patient was referred for duct ligation. She underwent PDA surgery on day 54 and the post-operative echocardiogram confirmed closure. Following PDA ligation, she was started on dexamethasone and extubated to non-invasive positive pressure ventilation (NIPPV) on day 108 and then weaned to humidified high flow nasal cannula oxygen (HHFNC) by day 114.

**Figure 1:**
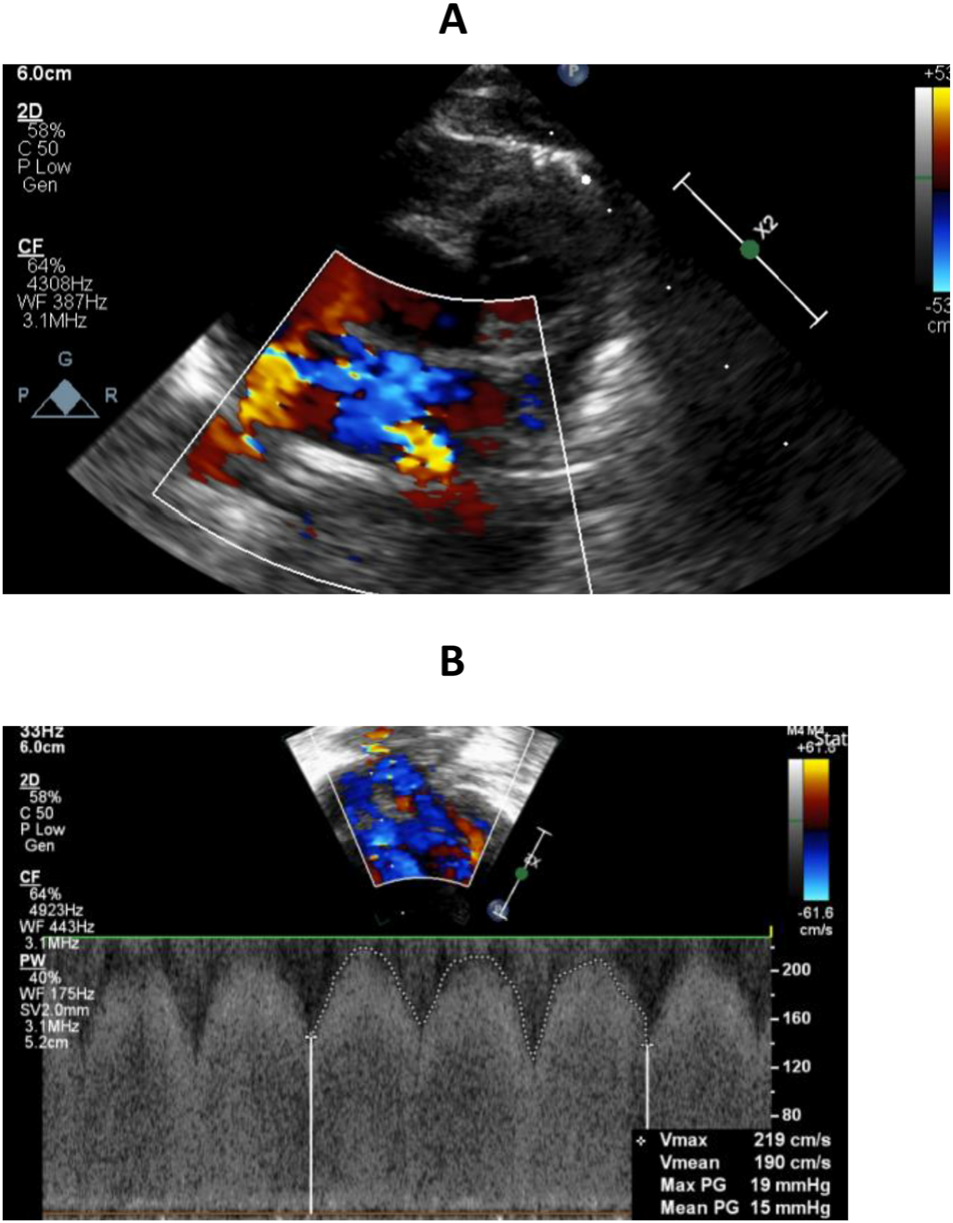
Pre dilation echo. (A) 2D echocardiography and colour Doppler of pulmonary veins demonstrating flow turbulence in lower pulmonary veins indicative of possible stenosis. (B) 2D echocardiography and pulse wave Doppler of pulmonary vein demonstrating increased velocity (2 m/s) and mean pressure gradient (15 mmHg). Note that the pulse waveform does not return to baseline, in keeping with features of an obstructed pulmonary vein.

**Figure 2:**
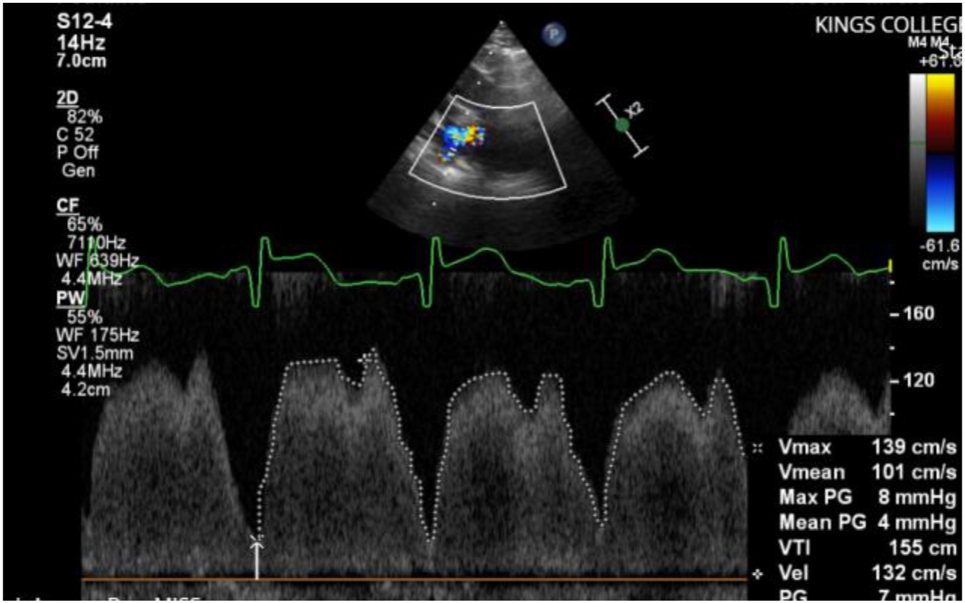
Post dilatation. 2D echocardiography and pulse wave Doppler of pulmonary vein demonstrating reduced velocity (1.39 m/s) and mean pressure gradient (4 mmHg) post dilatation. Note that the pulse waveform does return to baseline, in keeping with an unobstructed pulmonary vein.

On day 152 the patient suffered a respiratory deterioration. An echocardiogram demonstrated evidence of pulmonary hypertension with right heart dilation, septal flattening and suspected lower pulmonary vein stenosis (Figure 1A and B) with increased velocities up to 2 m/s (usually less than 0.7 m/s) and mean pressure gradient up to 15 mmHg (usually less than 4 mmHg). On day 167 another respiratory deterioration resulted in re-intubation. The patient was transferred for assessment of severe PVS with pulmonary hypertension. Cardiac computerised tomography (CT) demonstrated PV constriction and she underwent balloon catheter dilatation of the pulmonary veins and the atrial septum (to enlarge the vessels and septal defect and facilitate future intervention) on day 177. Following this her oxygen and ventilatory requirements were reduced and the patient was extubated to HHFNC within 48 h. Post-procedure, the flow velocity in the lower pulmonary veins had reduced to 1 m/s (Figure 2), the mean pressure gradient had reduced to 4 mmHg and there was left to right flow across the foramen ovale with mild tricuspid regurgitation (TR). Of note, the notched pulmonary artery on Doppler examination demonstrated increased pulmonary vascular resistance and poor compliance suggestive of ongoing pulmonary hypertension. The supplementary oxygen was weaned to 80 cc/min via nasal cannula. She was monitored with weekly echocardiograms.

On day 184 the infant had a further respiratory deterioration requiring support with HHFNC. Two weeks following balloon dilatation, an echocardiogram demonstrated recurrence of the PVS with an increased mean pressure gradient in the right pulmonary vein of 10  mmHg and in the left of 7  mmHg with an abnormal Doppler pattern. The infant was unable to maintain her oxygen saturations on HHFNC, therefore, she was re-intubated. An echocardiogram two weeks later demonstrated worsening of the PVS with evidence of severe pulmonary hypertension with an increased mean pressure gradient in the right lower pulmonary veins measuring 17 mmHg and left lower pulmonary vein measuring 10 mmHg with an abnormal pulse wave Doppler pattern and mild right ventricular dilation. There was right to left flow across the atrial communication and the TR velocity had increased to 4.8 m/s. Right and left lower PVS were confirmed which had likely contributed to the respiratory deterioration and a decision was made for further intervention. Unfortunately, due to capacity issues, the patient was transferred to a cardiac centre on day 220 for repeat cardiac catheterisation. During this admission, the patient underwent an additional balloon dilatation one month later following a further respiratory deterioration.

At 10 months of age, the infant underwent transcatheter stenting to open up both right and left pulmonary venous confluences. She was discharged home on supplementary oxygen, requiring intermittent hospital admission for respiratory decompensation and one further balloon dilatation of the stent at two years of age. Of note, she was started on systemic macrolide therapy with sirolimus following stent placement and remains stable on this.

## Discussion

The case we present highlights the recurrence of PVS in an extremely prematurely born infant who developed BPD and the utilisation of repeat echocardiograms to facilitate management.

It has been hypothesised that the inflammatory mechanisms, often secondary to prolonged mechanical ventilation such as those mediated by VEGF, which result in BPD and pulmonary hypertension may also contribute to PVS development postnatally [2]. PVS is typically diagnosed between 5 and 7.5 months of age often, as in our case, despite previous normal echocardiograms [1]. Frequently, the non-specific clinical presentation can result in delayed diagnosis whilst other more common clinical causes such as infection are ruled out. This is of particular importance in extremely prematurely born infants with established parenchymal lung disease whereby cardiovascular pathology may not be initially considered.

Earlier diagnosis between four and six months tends to occur in infants with cardiac defects, which may be attributed to an increased risk of PVS in this population or to more frequent echocardiographic monitoring. Premature infants are also at higher risk of having a PDA, which itself is a risk factor for PVS, with studies reporting a median prevalence of 50 % of infants with PVS also having a PDA diagnosis [2]. Investigation of a cohort of Trisomy 21 infants demonstrated that with each month of exposure to a left-to-right shunt the odds of PVS increased by 1.2 (95 % CI 1.06–1.39). The odds of developing PVS were even higher at 4.8 (95 % CI 1.4–16.8) amongst infants who were born at less than 35 weeks of gestation. Early closure of PDA, however, has not consistently been associated with improved outcomes or reduced PVS incidence, even in BPD cohorts. Clinicians may benefit in future from risk stratification scores to identify individuals at high risk of recurrent PVS [3].

Transthoracic echocardiography (TTE) remains the cornerstone of PVS evaluation, offering non-invasive haemodynamic assessment and facilitating early detection despite no standardised screening or diagnostic criteria [2]. Our case highlights the advantages of serial echocardiography, particularly in high-risk prematurely born infants. Indeed, a retrospective study of 213 neonates with severe BPD used serial review of echocardiograms to diagnose PVS and identify disease progression in 10 infants [4]. It is particularly important to ensure comprehensive TTE since Minich et al. demonstrated the likelihood of missing a PVS diagnosis on echo was eight times higher if only focussed echo was performed [5]. In this case, early diagnosis was made possible through neonatologists with European Association of Cardiovascular Imaging accreditation who were well-versed in performing comprehensive structural echocardiography and the support of a tertiary cardiac service. Clinicians may also benefit from screening guidelines for PVS in premature infants such as those outlined by the American Heart Association and the American Thoracic Society recommendations for evaluation of paediatric pulmonary hypertension, advocating for early evaluation in infants with severe respiratory distress syndrome or high oxygen requirements [6].

In total, our patient underwent three balloon dilatations prior to definitive transcatheter stenting at 10 months of age and one further stent dilatation at two years of age. Traditional vascular interventions such as catheter-interventions may not always effectively address recurrence and progression. Balloon dilatation is often a temporising measure, aimed at stabilising infants until they grow to a size appropriate for pulmonary vein stenting. To minimise complications such as clotting or stent stenosis, our cardiac centre generally will not perform stenting until effective dilatation of 6–7 mm. It is also anticipated that as infants grow, they may require further stent dilation. Some centres suggest surgical intervention as the first line treatment approach to increase early survival [7]. Infants treated via a surgical approach had higher survival rates and reduced need for re-intervention in a multi-centre study compared to initial catheter intervention [8]. However, the decision on which management approach to take must be on a clinical basis, with consideration of pre-operative co-morbidities, risks of open-heart surgery and bypass as well as local expertise.

Use of systemic therapies which modulate disrupted cellular pathways may prove more effective than catheter-based or surgical intervention alone. Emerging evidence suggests the use of systemic sirolimus, a macrolide, as adjunctive therapy in infants with severe PVS may be helpful, as demonstrated in our case. A study of 67 infants in the USA reported a significant survival advantage even when corrected for survival bias (p=0.027) compared to standard management [9]. Another study of 45 infants was able to demonstrate a significant reduction in intervention rates for PVS once participants were started on long term sirolimus therapy (5/year vs. 1.7/year) [10]. Small studies have shown promising results in improving one-year survival rates of congenital PVS through use of sirolimus-coated drug-eluting stents [11]. Long term therapy (median two years) has been shown to be well tolerated with no significant adverse outcomes [12].

In conclusion, our case of recurrent PVS in a 23-week gestation infant with BPD underscores the complexity of its pathogenesis and clinical management. We highlight the role of timely echocardiographic evaluation to recognise PVS in this population when presented with a clinical deterioration.

## Take-home message

Severe BPD poses a risk for the development of PVS in preterm infants. Diagnosis of PVS is easily missed due to the overlap of clinical symptoms with other conditions. Our case highlights that prompt echocardiographic evaluation upon clinical deterioration in infants with BPD could rule out PVS/PH. In future, serial echocardiographic screening protocols may be beneficial in this population.
